# DNA-BFN: Bayesian flow networks for generative modeling of regulatory DNA sequences

**DOI:** 10.1093/bioinformatics/btag785

**Published:** 2026-10-19

**Authors:** Han Yu, Qixiu Du, Lei Wei, Xiaowo Wang

**Affiliations:** Ministry of Education Key Laboratory of Bioinformatics, Center for Synthetic and Systems Biology, Beijing National Research Center for Information Science and Technology, Department of Automation, Tsinghua University, Beijing 100084, China; Ministry of Education Key Laboratory of Bioinformatics, Center for Synthetic and Systems Biology, Beijing National Research Center for Information Science and Technology, Department of Automation, Tsinghua University, Beijing 100084, China; Ministry of Education Key Laboratory of Bioinformatics, Center for Synthetic and Systems Biology, Beijing National Research Center for Information Science and Technology, Department of Automation, Tsinghua University, Beijing 100084, China; Ministry of Education Key Laboratory of Bioinformatics, Center for Synthetic and Systems Biology, Beijing National Research Center for Information Science and Technology, Department of Automation, Tsinghua University, Beijing 100084, China; College of Artificial Intelligence, Tsinghua University, Beijing 100084, China

## Abstract

**Motivation:**

Designing regulatory DNA sequences is challenging due to their discrete nucleotide alphabet and complex higher-order dependencies underlying regulatory function. Many existing generative models, particularly diffusion-based approaches, learn by injecting stochastic noise into sequences and training denoising processes, which can distort discrete sequence structure and weaken biological constraints.

**Results:**

Here, we introduce DNA Bayesian Flow Networks (DNA-BFN), a generative framework that models regulatory DNA sequences through iterative Bayesian updates of per-position nucleotide distributions. By directly modeling per-position categorical beliefs, DNA-BFN realizes a diffusion-like generative process without explicitly perturbing discrete nucleotide sequences, enabling fully differentiable training while preserving discrete sequence structure. Across eight regulatory sequence datasets spanning diverse species, scales, and sequence lengths, DNA-BFN consistently outperforms state-of-the-art baselines, faithfully preserves sequence feature statistics and regulatory patterns, enables few-shot sequence design, and achieves faster sampling efficiency. These results show that framing sequence generation as progressive uncertainty reduction in probability space provides an effective and generalizable approach for nucleotide sequence design.

**Availability and implementation:**

The software and its source code are publicly available at https://github.com/WangLabTHU/DNA-BFN. The dataset, source code and model weights are available in the Zenodo repository: https://doi.org/10.5281/zenodo.20177972.

## 1 Introduction

Regulatory DNA sequences are essential for controlling gene expression and form the basis of gene therapy, synthetic biology, and precision medicine (Levo and Segal 2014, Heinz *et al.* 2015, Moore *et al.* 2020, Donohue *et al.* 2022). Generating such sequences is a challenging generative modeling task: one must learn a distribution over high-dimensional discrete nucleotide sequences and sample high-quality candidates. The difficulty stems from strong nonlinear dependencies across positions and the extreme sparsity of functional sequences in the vast sequence space (Shrikumar *et al.* 2017, Lambert *et al.* 2018, Kim *et al.* 2021, Avsec *et al.* 2021b). When the true distribution is not well modeled, sampling often produces many biologically nonfunctional sequences, which undermines the reliability of sequence design (Li *et al.* 2025).

Early attempts at regulatory sequence generation relied on variational autoencoders (VAEs) and generative adversarial networks (GANs) (Wang *et al.* 2020, Seo *et al.* 2023, Zhang *et al.* 2023), but their continuous latent spaces and adversarial training often fail to preserve discrete regulatory patterns (e.g. motifs) and global dependencies. Diffusion models have recently become dominant in generative modeling. For regulatory DNA generation, diffusion can be performed in continuous latent or embedding spaces, enabling stable optimization and efficient training (Ho *et al.* 2020, DaSilva *et al.* 2026), but it requires discretization to map continuous representations back to nucleotide tokens, which introduces quantization error and a mismatch between the training objective and the sampling procedure. Alternatively, discrete diffusion model can be defined directly on discrete sequences (Hoogeboom *et al.* 2021, Du *et al.* 2024), where noise is added to nucleotide symbols; however, this may disrupt biological constraints such as motif integrity and spacing preferences (Georgakopoulos-Soares *et al.* 2023, Rauluseviciute *et al.* 2024, Feng *et al.* 2025), leading to distorted sequence structure and weakened biological constraints.

Bayesian Flow Networks (BFN) (Graves *et al.* 2023), as a promising approach, have shown strong performance in discrete data generation tasks (Song *et al.* 2024, Atkinson *et al.* 2025). BFN generates by iteratively updating beliefs over discrete tokens rather than operating directly on data, combining Bayesian inference with neural networks to realize a diffusion-like reverse generation process without explicitly constructing a forward process. This belief-updating formulation is naturally suitable for regulatory DNA sequence generation, as it updates beliefs over discrete bases rather than modifying the sequence directly. Compared with previous discrete diffusion methods, BFN benefits from differentiable inputs on the probability simplex, enabling gradient-guided sampling. These properties are advantageous for regulatory DNA sequence generation, because small discrete mutations can easily disrupt functional motifs and sequence grammar.

In this work, we present DNA Bayesian Flow Networks (DNA-BFN), a generative framework tailored for regulatory DNA sequence design. DNA-BFN models sequence generation as an iterative Bayesian updating process over per-position nucleotide distributions, operating directly in probability space. In contrast to diffusion-based approaches, it avoids explicit noise injection and instead performs progressive uncertainty reduction, which helps preserve discrete sequence structure and biological constraints. We evaluate DNA-BFN across a diverse set of regulatory sequence datasets and observe consistent improvements over existing generative models in terms of sequence feature statistics, and regulatory pattern preservation. In addition, DNA-BFN supports few-shot sequence design, enables the generation of sequences with enhanced functional properties, and achieves superior sampling efficiency compared with diffusion-based approaches. Together, these results suggest that Bayesian belief-based generative modeling provides an effective and generalizable framework for regulatory DNA sequence design.

## 2 Materials and methods

### 2.1 Deep generative models for DNA sequences

Designing regulatory DNA sequences is challenging because functional activity arises from combinatorial interactions among motifs and long-range dependencies, while the sequence space grows exponentially with length. Early deep generative models for DNA include VAEs and GANs, which have been widely applied to sequence generation and design (Wang *et al.* 2020, Seo *et al.* 2023, Zhang *et al.* 2023). VAEs learn an encoder–decoder mapping and optimize a variational lower bound, but the continuous latent representation and reconstruction objective often fail to preserve discrete motif structures and high-order dependencies. GANs, including Wasserstein GAN variants (WGAN) (Arjovsky *et al.* 2017), model sequence distributions via adversarial training, which can capture complex dependencies but suffers from unstable convergence and mode collapse, leading to reduced diversity and inconsistent biological validity.

Diffusion-based generative models have recently become prominent for DNA sequence generation owing to their powerful modeling capabilities (Li *et al.* 2024, DaSilva *et al.* 2026). These methods define a Markov forward process that progressively adds noise to sequences, and a learned reverse process that denoises toward the data distribution. For discrete DNA, diffusion can be implemented via multinomial diffusion (MDM) or dirichlet diffusion (DDSM), which operate directly on categorical distributions, or via continuous diffusion variants (e.g. PromoDiff) that diffuse in continuous embedding or simplex spaces (Avdeyev *et al.* 2023, Wang *et al.* 2023, Du *et al.* 2024). However, diffusion-based approaches rely on repeated stochastic noising and recovery steps (Graves *et al.* 2023); this repeated injection of noise can distort discrete sequence structure and weaken inherent biological constraints.

### 2.2 Bayesian flow networks

Bayesian Flow Networks define a generative framework in which information about an observed sample $x\in X^{L}$ is propagated through a sequence of stochastic, Bayesian-style updates over an intrinsic parameter θ, which encodes the distributional structure of x. This process admits a communication interpretation: a *sender* generates an observation by adding noise to the true data distribution and sampling from it, while a *receiver* refines its belief about θ via Bayesian inference, thereby progressively improving its predictions.

Each discrete sample is represented as a one-hot tensor $x\in {\left\{0,1\right\}}^{L\times K}$, where *L* indexes the set of discrete variables and *K* denotes the number of possible categorical states for each variable. Starting from an initialization $\theta^{\left(0\right)}\in R^{L\times K}$, BFNs perform *T* refinement steps i=1,2,…,T; at each step, given $\theta^{\left(i-1\right)}$, the model updates its belief using noisy sender samples.

1. **Sender (noisy observation).** A sender generates a noisy observation of the true data by sampling from a noisy distribution with accuracy $\alpha_{i-1}$, denoted $p_{s}(\cdot |x;\alpha_{i-1})$.
2. **Current predictive belief.** A neural network Φ maps the current parameter to a categorical belief over one-hot entries, yielding $\Phi (\theta^{\left(i-1\right)})$.
3. **Receiver distribution.** A receiver constructs its distribution by applying the same noise addition, producing $p_{r}(\cdot |\theta^{\left(i-1\right)})$.
4. **Match sender and receiver.** The model updates Φ by minimizing the discrepancy between $p_{s}$ and $p_{r}$, typically using Monte Carlo sampling to approximate expectations and accelerate training.
5. **Update the state.** Using the new observation from $p_{s}$ together with $\theta^{\left(i-1\right)}$, the intrinsic parameter is updated to $\theta^{\left(i\right)}$ via the BFN Bayesian update rule.

Formally, the loss function of BFNs can be written as


(1) $$L_{\text{BFN}}(x)=\sum_{i=1}^{T}\text{KL}(p_{s}(\cdot |x;\alpha_{i})\|p_{r}(\cdot |\theta^{\left(i-1\right)};\alpha_{i})).$$

By choosing an accuracy schedule ${\left\{\alpha_{i}\right\}}_{i=0}^{T}$ that increases with *i*, the sender distribution becomes progressively less noisy and approaches the true distributional pattern of x, enabling increasingly reliable estimation of θ.

After training, BFNs generate new sequences by obtaining the final receiver distribution $p_{r}(\cdot |\theta^{\left(T\right)})$ and sampling y from it, using the learned intrinsic distribution.

This sender–receiver formalism ensures that the network always operates on distribution parameters, even for discrete x, leading to a fully differentiable generative model without requiring an explicit forward noising process as in traditional diffusion models.

### 2.3 DNA Bayesian flow networks

As shown in Fig. 1, we now introduce DNA-BFN, a generative framework for regulatory DNA sequences. Let $X={\left\{A,T,C,G\right\}}^{L}$ denote DNA sequence space of length *L*, where $V=[v_{1},v_{2},v_{3},v_{4}]$ represents the ordering of {A,T,C,G} and K=4 is the vocabulary size (excluding special tokens).

**Figure 1:**
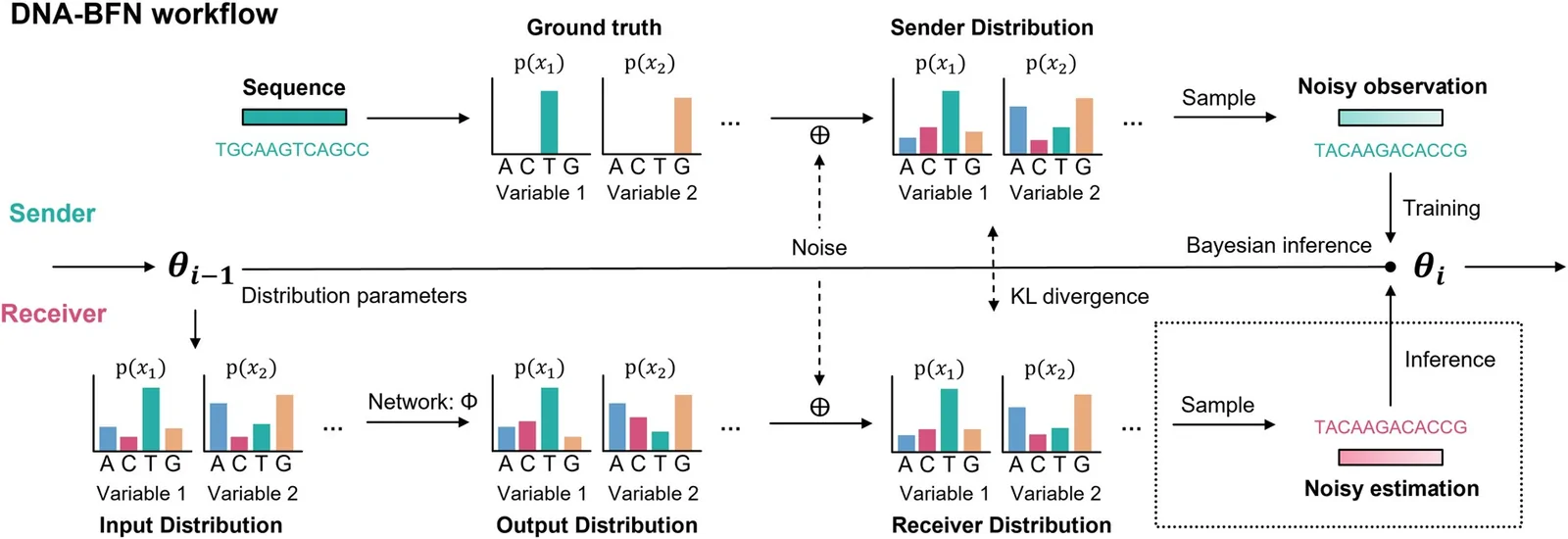
Overview of DNA-BFN. BFN uses Bayesian inference to update sequence distribution parameters from noisy observations, modeling each position as a categorical distribution over tokens. The network learns this noisy distribution during training and generates sequences by sampling from the predicted distribution during inference.

We view the generation process as a series of Bayesian updates with gradually increasing observation accuracy. At each step *i*, the model receives a noisy observation $y^{\left(i\right)}$ generated by a sender distribution, and a neural network Φ learns to approximate the distribution of the observation. This framework combines Bayesian inference and neural approximation to gradually recover the true DNA distribution.

A noisy observation at step *i*, $y^{\left(i\right)}=[y_{1}^{\left(i\right)},\ldots ,y_{L}^{\left(i\right)}]\in R^{L\times K}$, is drawn from the sender distribution:


(2) $$\begin{array}{c} p_{s}(y_{j}^{\left(i\right)}|x;\alpha_{i})=N(\alpha_{i}(Ke(x_{j})-1),\alpha_{i}KI),\\ p_{s}(y^{\left(i\right)}|x;\alpha_{i})=\prod_{j=1}^{L}\;p_{s}(y_{j}^{\left(i\right)}|x;\alpha_{i}), \end{array},$$

where $\alpha_{i}\in R^{+}$ is the accuracy at step *i*, and $e(x)\in R^{L\times K}$ is the one-hot encoding of x:


(3) $$e{\left(x\right)}_{i,j}=\{\begin{array}{cc} 1, & x_{i}=v_{j},\\ 0, & \text{otherwise} \end{array}.$$

By starting from some prior distribution $\theta^{\left(0\right)}$ over x and taking into account all observations so far, the input distribution is derived, providing the optimal estimation of the likelihood of each variable independently:


(4) $$\theta_{j,k}^{\left(i-1\right)}=\frac{\theta_{j,k}^{\left(i-2\right)}\;\text{exp}(y_{j,k}^{\left(i-1\right)})}{\sum_{q=1}^{K}\theta_{j,q}^{\left(i-2\right)}\;\text{exp}(y_{j,q}^{\left(i-1\right)})}=\frac{\theta_{j,k}^{\left(0\right)}\;\text{exp}\;\left(\sum_{p=1}^{i-1}y_{j,k}^{\left(p\right)}\right)}{\sum_{q=1}^{K}\theta_{j,q}^{\left(0\right)}\;\text{exp}\;\left(\sum_{p=1}^{i-1}y_{j,q}^{\left(p\right)}\right)}.$$

As $\theta^{\left(i-1\right)}$ is constructed under an independence assumption across $x_{1},\ldots ,x_{L}$, it is suboptimal when these variables are interdependent. The neural network Φ predicts the output distribution, which aims to provide a better estimation by considering interdependencies:


(5) $$\Phi {\left(\theta^{\left(i-1\right)}\right)}_{j,k}\approx p(x_{j}=v_{k}|y^{\left(1:i-1\right)}).$$

This approximation implies an equivalent approximation of the distribution of the next noisy observation $y^{\left(i\right)}$, referred to as the receiver distribution:


(6) $$\begin{array}{c} p_{r}(y_{j}^{\left(i\right)}|\Phi (\theta^{\left(i-1\right)});\alpha_{i})=\\ \sum_{k=1}^{K}\Phi {\left(\theta^{\left(i-1\right)}\right)}_{j,k}\;N(\alpha_{i}(Ke(v_{k})-1),\alpha_{i}KI)\\ p_{r}(y^{\left(i\right)}|\Phi (\theta^{\left(i-1\right)});\alpha_{i})=\\ \prod_{j=1}^{D}p_{r}(y_{j}^{\left(i\right)}|\Phi (\theta^{\left(i-1\right)});\alpha_{i}) \end{array}.$$

Specifically, at each step *i*, the error in Φ is measured through the KL-divergence between $p_{s}$ and $p_{r}$. For *T* steps,


(7) $$L^{T}=\sum_{i=1}^{T}D_{\text{KL}}(p_{s}(\cdot |x;\alpha_{i})\;\|\;p_{r}(\cdot |\Phi (\theta^{\left(i-1\right)});\alpha_{i})).$$

In the continuous-time limit T→∞, under a uniform prior $\theta^{\left(0\right)}$, the loss can be written as:


(8) $$L^{\infty }=E_{t∼U(0,1)}[E_{y^{\left(t\right)}∼p_{s}(\cdot |x;\beta (t))}[\frac{\alpha (t)}{2}\|e(x)-\Phi (\theta^{\left(t\right)}){\|}^{2}]],$$

where


(9) $$\beta (t)=\beta_{1}t^{2},\;\alpha (t)=\frac{d}{dt}\beta (t)=2\beta_{1}t.$$

The variable *t* is designed to transition from 0 to 1, and $\beta_{1}$ represents the artificially designed upper bound of the final accuracy at t=1.

#### 2.3.1 Entropy encoding

In the original BFN work (Graves *et al.* 2023), the current time step *t* is provided to the output network together with the input distribution θ(t). However, recent studies (Atkinson *et al.* 2025) have shown that during sampling, the entropy of θ(t) at a given *t* is typically higher than in training, likely due to accumulated uncertainty from iteratively using model output Φ(θ(t)). Consequently, traditional time-based Fourier encodings (where time *t* is an explicit input) fall outside the distribution of Φ(θ(t)), leading to poor performance. To address this issue, we follow previous work (Abramson *et al.* 2024, Atkinson *et al.* 2025) and replace the time-based Fourier encoding with an entropy-based Fourier encoding.

#### 2.3.2 Training

The training procedure of DNA-BFN follows the standard Bayesian Flow workflow (see Fig. 1) and is summarized in Supplementary Algorithm S1. Given a data sequence x∼D, we sample a time step t∼U(0,1) and set the accuracy $\alpha (t)=2\beta_{1}t$. A noisy observation $y^{\left(t\right)}$ is drawn from the *sender distribution* $p_{s}(y^{\left(t\right)}|x;\alpha_{t})$ (Eq. 2). This observation is used to perform a *Bayesian update* on the parameters of the input distribution:


$$\theta^{\left(t\right)}\leftarrow \mathrm{Softmax}(\sum_{p=1}^{t}y^{\left(p\right)}),$$

which corresponds to Eq. (4). The updated parameters $\theta^{\left(t\right)}$ (after entropy encoding) are fed into the neural network $\Phi_{\omega }$ to produce the parameters of the *output distribution* $\phi^{\left(t\right)}$. The continuous-time loss (Eq. 8), which is derived from the discrepancy between the *sender* and *receiver distributions*, takes the practical form of a weighted mean-squared error between e(x) and $\phi^{\left(t\right)}$.

#### 2.3.3 Inference

Sequence generation also follows the Bayesian flow process (Supplementary Algorithm S2). We start from the prior by setting $y^{\left(0\right)}=0$. For i=1,…,T, let t=(i−1)/T and s=i/T. At each step, the parameters of the current *input distribution* are obtained via *Bayesian update*, after which the network predicts the *output distribution* $\phi^{\left(t\right)}=\Phi_{\omega }(\mathrm{EntropyEncode}(\theta^{\left(t\right)}))$. A new noisy observation is drawn from the *sender distribution* centered on this prediction (Eq. 10):


(10) $$y^{\left(s\right)}=\beta (s)(K\phi^{\left(t\right)}-1)+\sqrt{K\beta (s)}z,\;z∼N(0,I).$$

After *T* steps, the final sequence is sampled from the categorical distribution induced by the converged parameters:


(11) $$x∼\mathrm{Categorical}\;(\mathrm{Softmax}(y^{\left(1\right)})).$$

### 2.4 Baseline models

We compare our method with several representative baseline models for regulatory sequence generation, covering statistical and adversarial models as reference baselines, and diffusion-based generative paradigms (Wang *et al.* 2020, 2023, Avdeyev *et al.* 2023, Du *et al.* 2024). PSSM represents a classical statistical baseline that models regulatory sequences using position-specific scoring matrix and generates new sequences by independent sampling at each position. WGAN applies the Wasserstein generative adversarial framework to promoter design, training a generator and a critic to minimize the Wasserstein distance between synthetic and natural sequence distributions, thereby improving training stability compared to standard GAN-based approaches. Multinomial Diffusion Model (MDM) formulates promoter design as a discrete diffusion process, where nucleotide sequences are progressively noised using a multinomial transition kernel and recovered through a learned reverse denoising process. Dirichlet Diffusion Score Model (DDSM) extends score-based diffusion to biological sequences by performing diffusion in the probability simplex via Dirichlet distributions, enabling continuous-time score matching while preserving categorical constraints during generation. Finally, PromoDiff is a diffusion-based deep generative model specifically developed for promoter engineering, which utilized UNet-based structures to embed sequences in a continuous space and generate functional promoters via diffusion. By integrating these diverse baseline models, we conduct a systematic comparison with our proposed approach to comprehensively evaluate its effectiveness.

### 2.5 Experiment setup

#### 2.5.1 Benchmark datasets

To comprehensively evaluate the performance and robustness of the proposed method across diverse regulatory sequence modeling scenarios, we construct a benchmark suite consisting of eight widely used regulatory sequence datasets, covering multiple species, sequence lengths, and dataset scales in Table 1. These datasets span multiple prokaryotic and eukaryotic systems, enabling systematic evaluation under varying levels of biological complexity and regulatory contexts.

**Table 1:** The regulatory sequence datasets for evaluation, spanning diverse data scales, species, and sequence lengths.

Name	Dataset	Data Scale	Species	Seq. Len.
**E-50**	Wang et al.	14 098	*E. coli*	50
**E-165**	Zhang et al.	13 972	*E. coli*	165
**H-200-H**	Agarwal et al.	10 000	Human	200
**H-200-K**	Agarwal et al.	10 000	Human	200
**H-200-W**	Agarwal et al.	10 000	Human	200
**F-500**	Taskiran et al.	6126	Fly	500
**H-500**	Taskiran et al.	3885	Human	500
**F-1001**	de Almeida et al.	1998	Fly	1001

Specifically, for short sequences, the benchmark includes two well-established *E. coli* promoter datasets, with sequence lengths ranging from 50 to 165 base pairs (bp) (Wang *et al.* 2020, Zhang *et al.* 2023). For mammalian regulatory elements, we used human datasets derived from lentiviral massively parallel reporter assays (lentiMPRA), filtering 10 000 cell-type–specific sequences of 200 bp for each of the HepG2, K562, and WTC11 lines (Agarwal *et al.* 2025). Moreover, for long-range sequences, we used cell-type–specific regulatory datasets from Drosophila Kenyon cells and human melanocytes (up to 500 bp) (Taskiran *et al.* 2024), as well as a more challenging Drosophila epidermis dataset with sequences exceeding 1 kb (de Almeida *et al.* 2024).

In summary, the benchmark covers dataset scales from under 2000 to over 10 000 samples and sequence lengths from 50 bp to over 1 kb. This diversity creates a rigorous testbed for assessing the generalization, scalability, and sequence-length sensitivity of generative models for regulatory sequence.

### 2.6 Evaluation metrics

We evaluate generated regulatory sequences using a set of complementary metrics that capture (i) sequence-level statistical features and (ii) biologically meaningful functional patterns.

#### 2.6.1 Sequence-level statistical features

The sequence-level statistics serve as fundamental descriptors that reflect the functional properties of regulatory sequences, as demonstrated in previous studies (Du *et al.* 2024). Let $S_{\text{nat}}$ and $S_{\text{gen}}$ denote the sets of natural and model-generated sequences, respectively.

- **GC-content distribution similarity (GC).** For each sequence *s*, define its GC content as the proportion of nucleotides *G* and *C* in the sequence. We compute the empirical GC content distributions, denoted as $P_{\text{nat}}$ for $S_{\text{nat}}$ and $P_{\text{gen}}$ for $S_{\text{gen}}$, using histogram binning, and then calculate the Jensen–Shannon divergence:
  $$\text{JS}(P_{\text{nat}},P_{\text{gen}})=\frac{1}{2}D_{\text{KL}}\left(P_{\text{nat}}\|M\right)+\frac{1}{2}D_{\text{KL}}\left(P_{\text{gen}}\;\|M\right),\;$$
   
  $$M=\frac{P_{\text{nat}}+P_{\text{gen}}}{2}.$$

Lower JS values indicate closer agreement in global nucleotide composition.

- **k-mer frequency consistency (k-mer).** For each *k*, let $f_{\text{nat}}^{\left(k\right)}$ and $f_{\text{gen}}^{\left(k\right)}$ denote the normalized frequency vectors of all observed k-mers in $S_{\text{nat}}$ and $S_{\text{gen}}$, respectively. We then measure their alignment using Pearson correlation:
  $$\rho^{\left(k\right)}=\text{PCC}\left(f_{\text{nat}}^{\left(k\right)},f_{\text{gen}}^{\left(k\right)}\right),\;k=4,\ldots ,9.$$

Higher $\rho^{\left(k\right)}$ indicates better preservation of local sequence motifs.

- **Edit distance (ED).** For each generated sequence $s\in S_{\text{gen}}$, compute the minimum Levenshtein distance to any natural sequence:
  $$d_{\text{min}}(s)=\underset{t\in S_{\text{nat}}}{\min}\text{Lev}(s,t),$$

and report the mean $\frac{1}{\left|S_{\text{gen}}\right|}\sum_{s}d_{\text{min}}(s)$. Smaller values indicate higher generative consistency.

#### 2.6.2 Biologically meaningful functional patterns

Biologically plausible regulatory sequences should not only match low-level sequence statistics, but also recapitulate functional signals that drive gene regulation. Therefore, we evaluate generated sequences to ensure they exhibit biologically meaningful patterns consistent with natural sequences.

- **Evo2-generated log-likelihood (LLD).** To assess whether the generated sequence $s\in S_{\text{gen}}$ conform to the biological constraints of natural sequences, we compute its log-likelihood under an oracle model (Brixi et al. 2025), the pretrained Evo2 DNA language model:
  $$\text{LLD}(s)=\frac{1}{\left|s\right|}\sum_{i=1}^{\left|s\right|}\;\text{log}\;p_{\text{Evo}2}(s_{i}|s_{<i}),$$

where $p_{\text{Evo}2}$ is the conditional nucleotide distribution learned by Evo2. We report mean LLD over all sequences in each dataset and model, where larger LLD values indicate higher biological plausibility.

- **Biological Functional Consistency (BFC).** To evaluate whether generated sequences preserve the functional patterns of natural sequences, we use pretrained functional genomic models (Enformer, Sei, and Borzoi) (Avsec et al. 2021a, Chen et al. 2022, Linder et al. 2025) to obtain cell-type–specific feature predictions for both natural and generated sequences. Let $X_{\text{nat}},X_{\text{gen}}\in R^{N\times C}$ denote the predicted feature matrices for natural and generated sequences, respectively, where *C* is the number of cell-type–specific features. To quantify whether the activation patterns across functional features are preserved, we compute:

1. **Wasserstein distance**
 
  $$W=\frac{1}{C}\sum_{j=1}^{C}W_{1}(X_{\text{nat}}^{\left(j\right)},X_{\text{gen}}^{\left(j\right)}),$$
  where $W_{1}$ is the 1-Wasserstein distance between the empirical distributions of feature *j*.
2. **Pearson correlation**
 
  $$\rho_{\text{func}}=\text{PCC}(\mu_{\text{nat}},\mu_{\text{gen}}),$$

where $\mu_{\text{nat}}=[\mu_{\text{nat}}^{\left(1\right)},\mu_{\text{nat}}^{\left(2\right)},\ldots ,\mu_{\text{nat}}^{\left(C\right)}]$, $\mu_{\text{gen}}=[\mu_{\text{gen}}^{\left(1\right)},\mu_{\text{gen}}^{\left(2\right)},\ldots ,\mu_{\text{gen}}^{\left(C\right)}]$, and each $\mu_{\text{nat}}^{\left(j\right)}$ and $\mu_{\text{gen}}^{\left(j\right)}$ denote the feature-wise mean predictions on $X_{\text{nat}}$ and $X_{\text{gen}}$, respectively.

Together, these metrics provide a multi-scale evaluation of both the statistical relevance and the biological plausibility of generated regulatory sequences.

## 3 Results

### 3.1 DNA-BFN–generated sequences better capture sequence features

We evaluate six generative models on multiple regulatory sequence datasets using three complementary metrics. As shown in Fig. 2a, DNA-BFN achieves the best or second-best performance across short sequence lengths (E-50, E-165). In contrast, diffusion-based models (MDM, DDSM) show superior performance on only a subset of metrics while generally yielding inferior results to DNA-BFN, validating the reliability and generability of DNA-BFN. The GAN-based model (WGAN), however, suffers from mode collapse, leading to poor performance. In addition, k-mer correlation analyses across different k values on the E-50 dataset show that DNA-BFN consistently outperforms the other generative models (Table S1, available as supplementary data at *Bioinformatics* online).

**Figure 2:**
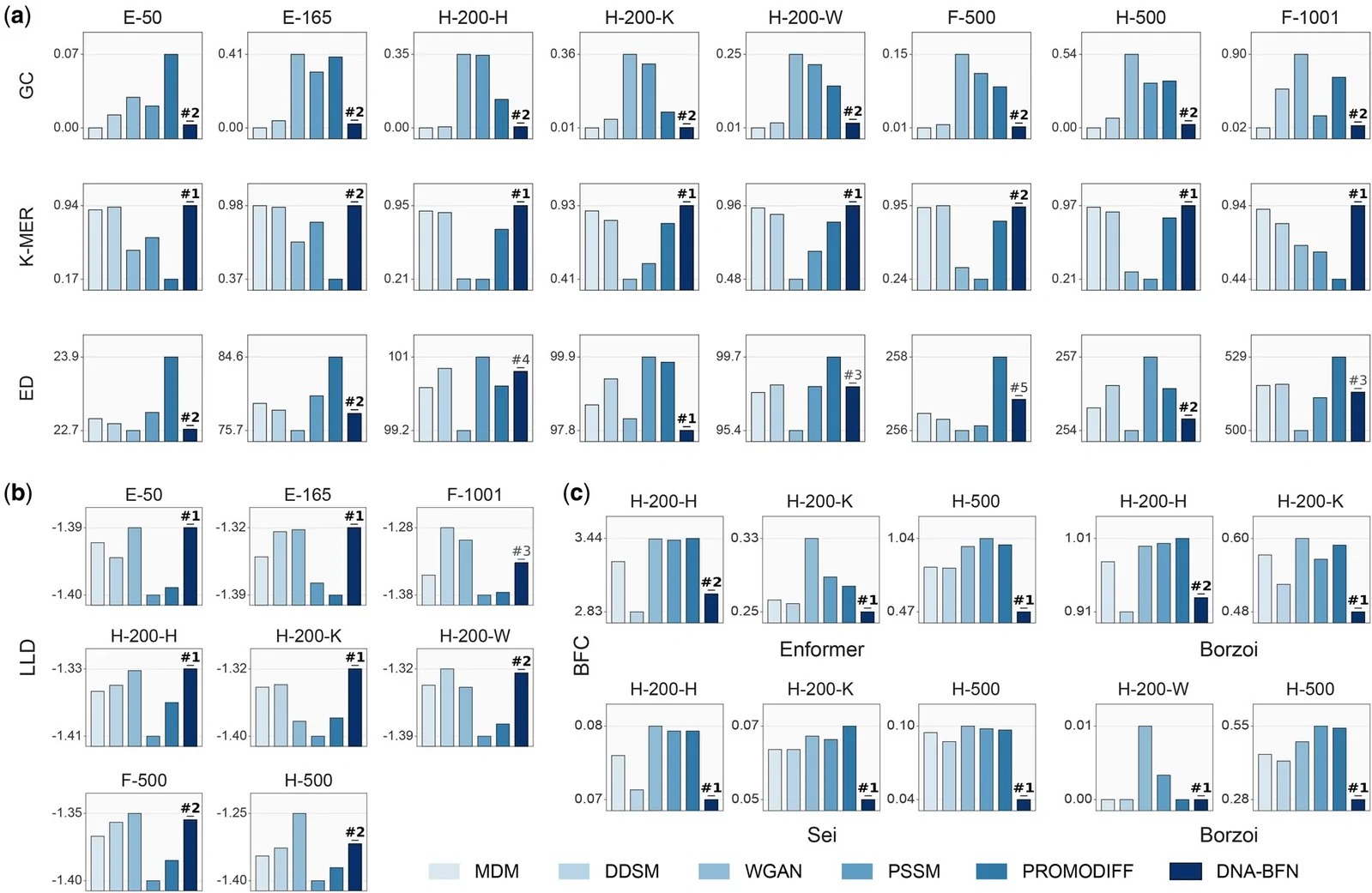
(a) Benchmarking of regulatory sequence generation models across eight datasets using three metrics: GC-content distribution similarity (GC), 6-mer frequency consistency (k-mer), and sequence ED. (b) Performance of different generative models evaluated by Evo2-generated log-likelihood across multiple datasets. (c) Mean Wasserstein distance between functional genomic model predictions (Enformer, Borzoi, and Sei) for generated versus natural sequences across four human-related datasets. Lower values indicate better agreement. For each metric, the best and second-best results are highlighted in bold.

On three 200 bp datasets (Fig. 2a), DNA-BFN again achieves the highest k-mer correlation across three human cell lines (HepG2, K562, and WTC11), obtains the best ED on K562, and remains highly competitive on HepG2 and WTC11. Notably, WGAN exhibits clear mode collapse on all three datasets, resulting in substantially lower k-mer correlations. Beyond unconditional generative models, we additionally compare with recently proposed conditional generative models for cell-type-specific regulatory sequence design on the three 200 bp datasets (DaSilva *et al.* 2026), and DNA-BFN achieves better generation performance (Table 2). For longer sequences (F-500, H-500, and F-1001, Fig. 2a), DNA-BFN consistently achieves the best or second-best k-mer correlation across all datasets, and ranks second in GC divergence.

**Table 2:** Benchmarking regulatory sequence generation models on 200 bp datasets using GC content JS divergence, k-mer correlation (*k* = 6), and average ED; DNA-diffusion denotes a conditional generative model.

Model	**H-200-H**			**H-200-K**			**H-200-W**		
	**GC (** ↓ **)**	**k-mer (** ↑ **)**	**ED (** ↓ **)**	**GC (** ↓ **)**	**k-mer (** ↑ **)**	**ED (** ↓ **)**	**GC (** ↓ **)**	**k-mer (** ↑ **)**	**ED (** ↓ **)**
**DNA-Diffusion**	0.1456	0.7025	98.84	0.0642	0.8278	99.77	0.0290	0.9553	97.48
**DNA-BFN**	0.0093	0.9515	100.34	0.0102	0.9332	97.79	0.0214	0.9603	97.94

To evaluate whether the generated sequences maintain diversity, we first analyzed the distribution of intra-sequence average EDs for sequences generated by different models. We found that, except for WGAN—which tends to suffer from mode collapse, leading to reduced diversity—other models, including DNA-BFN, maintained high diversity (Table S2, available as supplementary data at *Bioinformatics* online). Furthermore, BLAST searches against the natural genome indicate that DNA-BFN–generated sequences exhibit sufficient novelty and are not mere reproductions of genomic sequences, as shown in Fig. S1, available as supplementary data at *Bioinformatics* online.

To further investigate the sources of these performance gains, we performed ablation experiments on the E-50 dataset as a representative case. The results show that the Bayesian updating strategy and entropy encoding make complementary contributions to the overall performance of DNA-BFN (Table S3, available as supplementary data at *Bioinformatics* online).

Overall, DNA-BFN achieves the best or second-best results in 20 out of 24 metrics across the eight datasets, demonstrating strong capability and robustness in capturing sequence-level statistical features.

### 3.2 DNA-BFN captures biologically meaningful patterns

To further assess the biological validity of the generated sequences, we first evaluate six generative models using Evo2-generated log-likelihood scores (Brixi *et al.* 2025). As shown in Fig. 2b, DNA-BFN achieves the best or second-best performance on 7 out of 8 datasets. In particular, it obtains the highest log-likelihood on the E-50, E-165, H-200-H, and H-200-K datasets, and ranks second or remains close to the best on the H-200-W, F-500, and H-500 datasets. These results indicate that sequences generated by DNA-BFN better conform to the evolutionary constraints and functional patterns observed in real regulatory DNA. Notably, although WGAN obtains the best log-likelihood on several datasets, the generated sequences exhibit mode collapse, suggesting that these high scores may not reliably reflect robust biological patterns. In contrast, PSSM relies solely on position-wise probability sampling and therefore fails to capture higher-order dependencies, leading to substantially lower log-likelihoods. Other diffusion-based models achieve competitive results on some datasets but exhibit weaker global consistency than DNA-BFN.

We then compare the functional genomic prediction distributions of generated and natural sequences using three pretrained models (Enformer, Borzoi, and Sei) (Avsec *et al.* 2021a, Chen *et al.* 2022, Linder *et al.* 2025). Figure 2c reports the mean Wasserstein distance between model predictions on generated versus natural sequences across four human-related datasets. Across all datasets and prediction models, DNA-BFN consistently yields the lowest or second-lowest distances, indicating stronger alignment with the functional landscape of natural regulatory DNA. Notably, DNA-BFN achieves the best performance on Enformer and Borzoi across both H-200-K and H-200-W datasets, and substantially outperforms baselines on the H-500 dataset, where capturing long-range dependencies is particularly challenging. The superiority of DNA-BFN in Sei predictions further demonstrates its ability to preserve fine-grained regulatory signals. In contrast, PSSM and GAN-based approaches exhibit larger prediction discrepancies, suggesting weaker modeling of complex regulatory constraints.

Furthermore, as shown in Fig. 3a, we interpreted the predicted functional features on the H-500 dataset by comparing six generative models. DNA-BFN achieves the strongest agreement with natural sequences across all functional features, with a PCC of 0.991, indicating that the generated sequences faithfully preserve the regulatory feature landscape. In contrast, PSSM, WGAN, and PROMODIFF show weak correlations with natural sequences (PCC = 0.206, 0.227, and 0.264, respectively), with most predicted features compressed near zero, suggesting a substantial loss of functional signals. MDM performs moderately better (PCC = 0.489) but still exhibits clear deviations in functional space, while DDSM shows the best performance among the baseline methods (PCC = 0.751), although it continues to underestimate stronger regulatory signals. Across chromatin accessibility features (ATAC and DNase), promoter/enhancer marks (H3K4me3 and H3K27ac), and the SOX motif feature, DNA-BFN-generated sequences closely follow the natural-sequence trend, demonstrating its superior ability to capture multi-layer regulatory grammar and context-dependent functional patterns.

**Figure 3:**
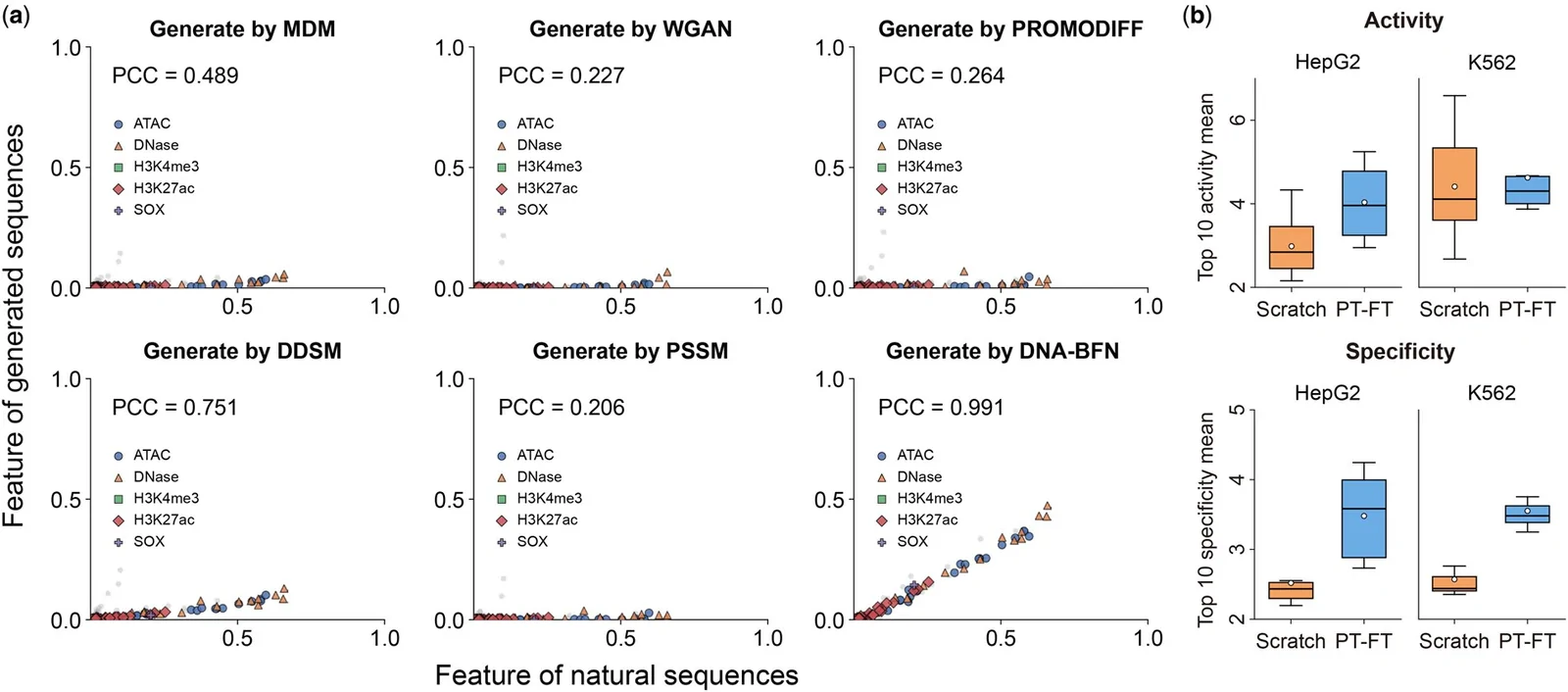
(a) Mean distribution of functional features in melanocyte cells (H-500) for sequences generated by six models and for natural sequences. Five representative feature types are highlighted in different colors. (b) Predicted activity and specificity for the top 10 HepG2- and K562-specific sequences under different design strategies, shown as box plots. Scratch denotes training from scratch, and PT–FT denotes pretraining followed by fine-tuning.

To further evaluate motif-level regulatory grammar, we performed motif-pair analysis using Polygraph (Lal *et al.* 2025). A total of 1191 motif pairs were selected from the H-500 training set, and their occurrence, orientation, and spacing patterns were compared between generated and natural sequences. DNA-BFN showed only one differential motif pair in occurrence, substantially fewer than the baseline methods. It also achieved the lowest number of differential orientation pairs (six) and the lowest number of differential spacing pairs (29). These results indicate that DNA-BFN more closely preserves the motif-pair composition and spatial organization (Table S4, available as supplementary data at *Bioinformatics* online).

Overall, DNA-BFN not only reproduces low-order sequence statistics but also recapitulates global functional constraints in high-dimensional functional space, generating sequences that closely match native regulatory patterns.

### 3.3 A few-shot strategy for cell-type-specific sequence design

Sequence feature statistics and biologically meaningful pattern recovery demonstrate that DNA-BFN is a reliable generative model. In practice, a challenging task is to generate cell-type-specific regulatory sequences with very few available training samples. To address this low-data setting, we introduce a pretraining–finetuning strategy for DNA-BFN. The model is first pretrained on publicly available 200 bp human DNase I hypersensitivity datasets covering HepG2, K562, and WTC11, enabling it to learn general regulatory sequence features associated with open chromatin across multiple cellular contexts. It is then finetuned using only 100 known cell-type-specific sequences for HepG2 and K562 (Agarwal *et al.* 2025). This strategy allows DNA-BFN to transfer broadly learned regulatory patterns from large-scale chromatin accessibility data while adapting efficiently to the sequence features that drive cell-type-specific activity.

To evaluate whether the generated sequences exhibit both functional activity and cell-type specificity, we use an independent MPRA activity predictor as an external oracle (Gosai *et al.* 2024). Because an independent predictor for WTC11 is unavailable, WTC11 is included only in the pretraining stage and excluded from evaluation. As shown in Fig. 3b, the pretraining–finetuning strategy markedly improves the top-10 mean predicted specificity for both HepG2 and K562, while also increasing predicted activity. These results suggest that DNA-BFN can use a small number of cell-type-specific examples to guide generation toward sequences that are not only regulatory-like, but also preferentially active in the desired cellular context. Overall, the few-shot design results demonstrate the practical potential of DNA-BFN for scalable cell-type-specific inverse design of regulatory DNA sequences.

### 3.4 Sampling efficiency of DNA-BFN

Sampling efficiency is an important consideration when applying generative models to large-scale inverse DNA sequence design, especially in scenarios where a large number of candidate sequences need to be generated and screened. Table 3 compares the time required by different generative models to generate 10 000 samples across eight datasets. Although WGAN and PSSM achieve lower absolute sampling times, the quality of their generated sequences is generally unsatisfactory, which limits their practical utility in real design tasks. In contrast, the comparison between DNA-BFN and diffusion-based models, including MDM, DDSM, and PROMODIFF, is more meaningful, as these methods also rely on iterative sampling procedures and aim to generate higher-quality sequences.

**Table 3:** Time consumption (in seconds) for generating 10 000 samples across eight datasets using different generative models.

Model	E-50	E-165	H-200-H	H-200-K	H-200-W	F-500	H-500	F-1001
**MDM**	280	1044	1621	1617	1620	2572	2573	4570
**DDSM**	35	78	173	173	172	334	408	1693
**WGAN**	3	9	13	11	17	35	43	286
**PSSM**	7	24	30	30	58	74	73	177
**PROMODIFF**	182	387	358	358	359	701	702	2368
**DNA-BFN**	10	29	82	85	84	113	112	516

As shown in the results, DNA-BFN is substantially faster than diffusion-based baselines across all datasets. For example, on the E-50 dataset, DNA-BFN requires only 10 s to generate 10 000 samples, whereas MDM, DDSM, and PROMODIFF require 280, 35, and 182 s, respectively, corresponding to speedups of 28.0×, 3.5×, and 18.2×. Similar efficiency advantages are also observed on longer-sequence datasets: on F-500 and H-500, DNA-BFN requires 113 and 112 s, respectively, while MDM requires 2572 and 2573 s, PROMODIFF requires 701 and 702 s, and DDSM requires 334 and 408 s. On the longest F-1001 dataset, DNA-BFN still completes the generation of 10 000 samples within 516 s, substantially reducing the sampling cost compared with MDM, DDSM, and PROMODIFF, which require 4570, 1693, and 2368 s, respectively. This advantage remains stable as sequence length increases. Overall, these results demonstrate that DNA-BFN can significantly reduce sampling cost while maintaining comparable or better generation quality, providing an efficient and scalable solution for large-scale, high-quality DNA sequence generation.

## 4 Conclusion

Generating functional regulatory DNA sequences is a fundamental challenge in synthetic biology, primarily due to the discrete nature of genomic data and the complex, higher-order grammar governing gene expression. Traditional diffusion-based models often rely on injecting stochastic noise into sequences, which can inadvertently distort delicate nucleotide constraints and disrupt essential regulatory architectures. In this study, we introduced DNA-BFN, a generative framework that shifts the paradigm from noise-injected denoising to progressive uncertainty reduction within a Bayesian flow. By operating directly on categorical beliefs in a continuous probability space, DNA-BFN provides a fully differentiable path for sequence generation that respects the discrete structure of DNA, enabling precise design across diverse species and functional contexts.

Our results demonstrate that DNA-BFN effectively captures the regulatory grammar of DNA, from k-mer frequencies to complex transcription factor binding dependencies. Unlike previous models that often suffer from mode collapse or generate biologically implausible sequences, DNA-BFN maintains high biological fidelity. Across eight datasets, DNA-BFN consistently matched the statistical distributions of natural sequences and exhibited high correlation across different functional channels. This suggests the model does not merely memorize training data but learns the underlying syntax of regulatory DNA.

A key contribution of this work is the few-shot strategy for cell-type-specific design. By fine-tuning the pre-trained DNA-BFN with limited samples, we generated synthetic elements with high specificity for targets such as HepG2 and K562. The ability to generate sequences with high predicted activity and specificity—even with sparse data—highlights DNA-BFN’s potential as a modular tool for programmable gene control. This efficiency is particularly valuable for designing synthetic promoters or enhancers for rare cell types or specific disease states where large-scale training data is unavailable.

Despite these advances, several limitations remain. First, while in silico validation using transformer-based experts provides strong evidence of activity, these predictions are proxies for biological behavior. Future work must incorporate high-throughput experimental assays, such as MPRAs, to validate the functional efficacy of designed sequences. Second, the current benchmarks contain regulatory DNA sequences of up to 1001 bp and therefore do not directly evaluate dependencies spanning several kilobases or longer genomic distances. Although the dense self-attention architecture of DNA-BFN provides a global receptive field, extending the current implementation to substantially longer contexts incurs rapidly increasing computational and memory costs. Evaluating DNA-BFN on long-context regulatory benchmarks and incorporating more efficient long-context architectures therefore represent important directions for future work. Third, although the primary benchmarks use random splits of genomic sequences, we additionally performed a homology-controlled evaluation on the E-50 dataset using MMseqs2-based clustering with an 80% sequence identity threshold to construct de-redundant training and test sets. Under this more stringent setting, the relative performance trends across models remained consistent with the original evaluation (Table S5, available as supplementary data at *Bioinformatics* online). Extending such homology-aware evaluation to all benchmark datasets would provide a more comprehensive assessment of model generalization. Fourth, although our model captures complex dependencies, the “black box” nature of deep generative architectures limits our ability to explicitly extract the learned regulatory logic or causal relationships between specific nucleotide motifs.

Looking forward, DNA-BFN provides a mathematically rigorous platform for the de novo design of the non-coding genome. Integrating this framework with multi-modal data, such as epigenetic marks and protein-DNA interaction profiles, could further refine design capability. Expanding the model to handle multi-task optimization—simultaneously targeting activity, tissue-specificity, and environmental responsiveness—will move us closer to fully programmable transcriptional networks. These developments will accelerate the design of precise genetic medicines and context-aware synthetic biological systems, advancing both our understanding of gene regulation and its practical applications.

## Supplementary Material

btag785_Supplementary_Data

## Data Availability

The software and its source code are publicly available at https://github.com/WangLabTHU/DNA-BFN. The dataset, source code and model weights are available in the Zenodo repository: https://doi.org/10.5281/zenodo.20177972.
