# Supplementary material for: DNA-BFN: Bayesian flow networks for generative modeling of regulatory DNA sequences
Source: Bioinformatics. 2026 Oct 19;42(10):btag785. doi: 10.1093/bioinformatics/btag785 (PMC13721123; doi:10.1093/bioinformatics/btag785)
Supplement: Supplementary file 1 [file btag785_supplementary_data.pdf]

Supplementary Information for
DNA-BFN: Bayesian Flow Networks for Generative Modeling of Regulatory DNA Sequences

Han Yu, Qixiu Du, Lei Wei, and Xiaowo Wang

Appendix

DNA-BFN Model Architecture

The DNA-BFN model is implemented as a Transformer encoder, augmented with entropy-aware input representations and rotary position-aware self-attention. The model has been deployed on the previously developed GPro platform (Wang et al., 2024) available at https://github.com/WangLabTHU/GPro for one-click usage.

Input Augmentation.

Given an input sequence $\boldsymbol{\theta} \in \mathbb{R}^{L\times D}$, where each position represents a categorical probability distribution, we compute the normalized Shannon entropy

$$h_i = \frac{-\sum_{j=1}^{D} \theta_{ij} \log \theta_{ij}}{\log D}. \tag{1}$$

The entropy value is mapped to a Fourier feature representation $\phi(h_i)$ and concatenated with the original input:

$$\mathbf{x}_i = [\boldsymbol{\theta}_i; \phi(h_i)]. \tag{2}$$

Embedding and Encoder.

The augmented input is linearly projected and normalized before being processed by a stack of N Transformer blocks. Each block follows a Pre-LayerNorm structure:

$$\tilde{\mathbf{h}}^{(l)} = \mathbf{h}^{(l)} + \mathrm{MHSA}\Big(\mathrm{LN}\Big(\mathbf{h}^{(l)}\Big)\Big), \tag{3}$$

$$\mathbf{h}^{(l+1)} = \tilde{\mathbf{h}}^{(l)} + \mathrm{FFN}\Big(\mathrm{LN}\Big(\tilde{\mathbf{h}}^{(l)}\Big)\Big), \tag{4}$$

where MHSA denotes dense multi-head self-attention and FFN denotes a position-wise feed-forward network with GELU activation.

Rotary Position Encoding.

For each attention head, rotary positional embeddings are applied to the query and key vectors prior to attention computation:

$$\tilde{\mathbf{q}} = \mathrm{RoPE}(\mathbf{q}), \quad \tilde{\mathbf{k}} = \mathrm{RoPE}(\mathbf{k}), \tag{5}$$

followed by standard scaled dot-product attention

$$\mathrm{Attn}(\mathbf{Q}, \mathbf{K}, \mathbf{V}) = \mathrm{softmax}\left(\frac{\tilde{\mathbf{Q}}\tilde{\mathbf{K}}^\top}{\sqrt{d}}\right)\mathbf{V}. \tag{6}$$

Output Head.

The final representation is mapped to the output space using a RoBERTa-style prediction head:

$$\mathbf{y} = W_2\,\mathrm{LN}\Big(\sigma(W_1\,\mathrm{LN}(\mathbf{h}^{(N)}))\Big), \tag{7}$$

where $\sigma(\cdot)$ denotes the GELU nonlinearity.

Implementation Details.

DNA-BFN was implemented in JAX using Haiku and Optax. The model uses a 6-layer Pre-LayerNorm Transformer encoder with an embedding dimension of 512, a feed-forward hidden dimension of 512, and 8 attention heads, corresponding to a head dimension of 64. Rotary positional embeddings are applied to the query and key representations. The input categorical distribution is augmented with entropy-based Fourier features before being projected into the Transformer embedding space. The final representation is mapped to the output space using a RoBERTa-style prediction head.

The model contains approximately 9.75 million trainable parameters under the default configuration. Training is performed using the Adam optimizer with $\beta_1 = 0.9$ and $\beta_2 = 0.999$, a learning rate of 1×10^{-4}, a batch size of 16, and 20,000 optimization steps. The random seed is fixed to 42, and each dataset is randomly partitioned into training and evaluation sets at a ratio of 90:10, consistent with previous work (Du et al., 2024). The BFN final precision parameter is set to $\beta_1 = 2.0$. The vocabulary consists of six special tokens, <unk>, <pad>, <mask>, <cls>, <eos>, <bos>, and the four DNA bases A, T, C, G. We use 50 Monte Carlo samples for the randomized continuous-time loss estimator, except for the 1001-bp dataset, where 10 samples are used to reduce computational cost. No dropout, weight decay, or gradient clipping is used. Evaluation is performed every 100 training steps, and model checkpoints are saved every 5,000 steps. All experiments were conducted using an NVIDIA L40S GPU. Unless otherwise specified, the same architecture and training settings are used across all datasets.

Evaluation Setup.

All metrics were computed by comparing generated sequences with natural sequences. GC and k-mer metrics were evaluated using 10,000 generated sequences, while for ED and functional metrics (LLD and BFC), a random subset of 100 generated sequences was used to reduce computational cost.

Generative strategies for Regulatory sequences

Multinomial Diffusion Model. In MDM, one-hot representations are directly noised via a multinomial forward diffusion process, in which the standard Gaussian noise addition is replaced by a corresponding uniform noise process:

$$q(\mathbf{x}^{(i)} \mid \mathbf{x}^{(i-1)}) = \mathscr{C}(\mathbf{x}^{(i)} \mid (1-\beta_i)\mathbf{x}^{(i-1)} + \beta_i/K)$$

Analogous to classical diffusion models, the posterior $q(\mathbf{x}^{(i-1)} \mid \mathbf{x}^{(i)}, \mathbf{x}^{(0)})$ remains analytically tractable even in the discrete (multinomial) case. Therefore, the parameters θ of the generative reverse process can be optimized by minimizing the Kullback–Leibler divergence between $p_\theta(\mathbf{x}_{i-1} \mid \mathbf{x}^{(i)})$ and $q(\mathbf{x}^{(i-1)} \mid \mathbf{x}^{(i)}, \mathbf{x}^{(0)})$:

$$\mathcal{L}_{\mathrm{MDM}}(\mathbf{x}) = \sum_{i=1}^{T} \mathrm{KL}\Big(p_\theta(\mathbf{x}^{(i-1)} \mid \mathbf{x}^{(i)}) \,\big\|\, q(\mathbf{x}^{(i-1)} \mid \mathbf{x}^{(i)}, \mathbf{x}^{(0)})\Big)$$

Training is performed with batch size 64 and learning rate 10^{-4}. The model employs a 12-layer transformer architecture with 16 attention heads per layer. Diffusion is performed over 100 timesteps. The local attention window size is chosen to be a divisor of the input sequence length (e.g., 25 for 50-bp sequences in the E-50 dataset). Default training lasts 100 epochs on a 90/10 train/validation split.

Dirichlet Diffusion Score Model. DDSM maps discrete variables into the continuous probability simplex using the Dirichlet distribution, performs diffusion therein, and maps back to discrete space. More specifically, DDSM first projects the traditional K-dimensional one-hot embedding $\mathbf{x} \in \{0,1\}^K$ (with $\sum_{k=1}^{K} x_k = 1$) into a $(K-1)$-dimensional space $\mathbf{v} \in (0,1)^{K-1}$ via the classical stick-breaking construction:

$$\mathbf{x}_1^{(i)} = \mathbf{v}_1^{(i)}, \mathbf{x}_2^{(i)} = (1-\mathbf{v}_1^{(i)})\mathbf{v}_2^{(i)}, \mathbf{x}_3^{(i)} = (1-\mathbf{v}_1^{(i)})(1-\mathbf{v}_2^{(i)})\mathbf{v}_3^{(i)} \ldots$$

The resulting $\mathbf{v} \in (0,1)^{K-1}$ undergoes a multivariate Jacobi diffusion process (equivalent to independent univariate diffusions per coordinate under reparameterization), progressively noising $v_1, v_2, \ldots, v_{K-1}$ toward $\mathrm{Beta}(1, K-1), \mathrm{Beta}(1, K-2), \ldots, \mathrm{Beta}(1, 1)$, respectively:

$$d\mathbf{v} = \frac{1}{2}[a(1-\mathbf{v}) - b\mathbf{v}]dt + \sqrt{\mathbf{v}(1-\mathbf{v})}d\mathbf{w}$$

where $\mathbf{w}$ is the standard Wiener process, and a, b are the parameters of targeted Beta distribution. Then the reverse denoising process can be formulated as:

$$d\mathbf{v} = \Big\{\frac{1}{2}[a(1-\mathbf{v}) - b\mathbf{v}] - (1-2\mathbf{v}) - \mathbf{v}(1-\mathbf{v})\nabla_{\mathbf{v}} \log p_t(\mathbf{v})\Big\}dt + \sqrt{\mathbf{v}(1-\mathbf{v})}d\bar{\mathbf{w}}$$

The model parameters are learned by replacing $\nabla_{\mathbf{v}} \log p_t(\mathbf{v})$ with the final denoising model; samples in $\mathbf{v}$-space are then mapped back to the simplex via the inverse stick-breaking transform, yielding valid categorical samples equivalent to Dirichlet draws.

Training is performed with batch size 64 and learning rate 5×10^{-4}. The score network is a dilated 1D U-Net (20 residual blocks, 256 channels, kernel size 9) conditioned on Gaussian Fourier timestep embeddings. Dilations follow the pattern [1,4,16,64] in symmetric arrangement. Diffusion employs 400 steps. Default training lasts 100 epochs on a 90/10 train/validation split. Sequences are sampled via the Euler–Maruyama method.

Diffusion Models based on UNet. Both PromoDiff and DNA-Diffusion models exemplify the canonical paradigm of applying diffusion models to discrete sequence design. Specifically, they employ a UNet-like architecture to process the representation of $\mathbf{x}$, followed by the standard diffusion process in continuous space. Within this framework, both the forward process and the objective function align with those of the denoising diffusion probabilistic model (DDPM):

$$q(\mathbf{x}^{(i)} \mid \mathbf{x}^{(i-1)}) = N(\mathbf{x}^{(i)} \mid (1-\beta_i)\mathbf{x}^{(i-1)}, \beta_i \mathbf{I})$$

$$\mathcal{L}_{\mathrm{PromoDiff/DNA-Diffusion}}(\mathbf{x}) = \sum_{i=1}^{T} \mathrm{KL}\Big(p_\theta(\mathbf{x}^{(i-1)} \mid \mathbf{x}^{(i)}) \,\big\|\, q(\mathbf{x}^{(i-1)} \mid \mathbf{x}^{(i)}, \mathbf{x}^{(0)})\Big)$$

For PromoDiff, training is performed with batch size 64 and learning rate 10^{-4}. Following standard DDPM, the diffusion model uses a linear β-schedule from 10^{-4} to 0.02 over 100 timesteps. Generated samples are post-processed by clamping to $[-1, 1]$, rescaling to $[0, 1]$, and converting to uint8 representation. Default training lasts 100 epochs on a 90/10 train/validation split.

For DNA-Diffusion, training is performed with batch size 120 and learning rate 10^{-4}. The diffusion schedule is linear (β from 10^{-4} to 0.2, 50 steps). The U-Net backbone has base dimension 200 and multipliers $[1, 2, 4]$, with timestep conditioning via learned sinusoidal positional embeddings projected to dimension 800. Each stage includes ResNet blocks (groups=4) and linear self-attention. Default training lasts 100 epochs on a 90/10 train/validation split.

Wasserstein Generative Adversarial Models Generative Adversarial Networks (GANs) consist of two competing networks: a generator G and a discriminator D. The generator aims to approximate the true data distribution p_r by producing realistic synthetic samples, while the discriminator seeks to distinguish real samples from generated ones. This adversarial process is formulated as the following minimax objective:

$$\min_G \max_D V(D, G) = \mathbb{E}_{x\sim p_r}[\log D(x)] + \mathbb{E}_{z\sim p_z}[\log(1 - D(G(z)))].$$

In WGANs, the Wasserstein distance serves as the objective, with the discriminator constrained to 1-Lipschitz functions. This yields more stable training and a loss that correlates more reliably with generated sample quality.

Training is performed with batch size 32 and learning rate 10^{-4} for both generator and discriminator. The generator takes 128-dimensional Gaussian noise, applies a linear projection, followed by five residual blocks (kernel size 5) and a 1×1 convolution with softmax activation to output nucleotide probabilities. The discriminator processes one-hot sequences through an initial Conv1d (kernel size 5, 512 channels), five residual blocks, and a linear layer producing a scalar score. We saved checkpoints every epoch (total 12) and selected the best-performing one for sampling. Default training runs on a 90/10 train/validation split.

Position-Specific Scoring Matrix As a simple yet informative baseline, we constructed a position-specific scoring matrix (PSSM) directly from the training dataset by computing the empirical frequency (occurrence probability) of each nucleotide at each position. Sequences were then generated by independently sampling nucleotides according to these position-wise probabilities.

$$P(\mathbf{x}_i = b) = \frac{\text{count of nucleotide } b \text{ at position } i}{\text{total number of sequences}}, \quad b \in \{\mathrm{A}, \mathrm{C}, \mathrm{G}, \mathrm{T}\},$$

This approach represents the most straightforward frequency-based generative model that captures only positional marginal

distributions, ignoring any higher-order dependencies. If a learned generative model fails to outperform this PSSM-based sampler in downstream evaluation metrics, it can be considered ineffective, as it does not learn meaningful sequence patterns beyond what is already achievable by independent per-position sampling.

Pretraining–Finetuning Strategy and Evaluation Protocol

To address the scarcity of experimentally validated high-activity and high-specificity sequences, we adopt a two-stage pretraining–finetuning strategy. The overall workflow consists of (i) constructing a large-scale pretraining dataset, (ii) selecting cell type–specific sequences for finetuning.

Pretraining dataset.

We pretrain the model on publicly available 200 bp human DNase I hypersensitivity (DHS) datasets. DHS sequences from HepG2, K562 and WTC11 merged and deduplicated, yielding a total of 341,320 sequences (Agarwal et al., 2025). This pretraining phase enables the model to learn general sequence regulatory principles across multiple cell types, thereby providing a strong initialization for downstream specificity-oriented finetuning.

Cell type–specific metric.

To ensure that finetuning focuses on sequences with strong cell-type specificity, we select a specificity-aware model Malinois as criterion (Gosai et al., 2024). Malinois is a CNN-based predictor trained on over 700,000 regulatory sequences to assess regulatory potential in three cell types (HepG2, K562, SK-N-SH). We implemented the model using the pretrained weights and followed the official repository's guidelines for environment setup (`https://github.com/sjgosai/boda2`).

For each target cell type t, let $a_t(s)$ denote the predicted activity (e.g., $\log_2$ fold change) of sequence s in cell type t, and let $\{a_c(s)\}_{c \neq t}$ denote predicted activities in all non-target cell types. We define the *minGap* specificity score as:

$$\text{minGap}(s,t) = \min_{c \neq t} \left(a_t(s) - a_c(s) \right).$$

minGap measures the smallest margin by which a sequence is more active in the target cell type than in any other cell type. A larger minGap thus indicates stronger and more robust cell-type specificity.

Training strategies.

We compare two training strategies:

(i) Training from scratch. The model is trained directly on the 100 selected sequences for each target cell type without any pretraining, for 500 steps.

(ii) Pretraining–finetuning. The model is first pretrained on the 341,320 DHS sequences using the same configuration as in previous experiments. The pretrained model is then finetuned on the 100 cell type-specific sequences for 500 steps. The training configuration remains unchanged between pretraining and finetuning, except for the number of training steps.

Generation and evaluation.

For each model, 10,000 sequences are generated. An independent MPRA activity predictor is used as an external oracle to assess functional activity. The activity of a sequence in a specific cell type t is defined as

$$a_t(s),$$

where s denotes the generated sequence. For each generated sequence, we compute the specificity score:

$$\text{Spec}(s,t) = a_t(s) - \max_{c \neq t} a_c(s).$$

This score quantifies how much more active a sequence is in the target cell type than in any non-target cell type. The top 10 sequences with the highest specificity scores are selected for detailed analysis. Their activity and specificity are reported as representative performance metrics. WTC11 is excluded from evaluation due to the lack of an independent predictor.

Functional genomic models for Biological-Level Evaluation

Evo2 We accessed the Evo2-40B API provided by NVIDIA (`https://build.nvidia.com/arc/evo2-40b`). Since Evo2 is a genomic language model and does not inherently score the function variants, we computed the log-likelihood of the entire sequence distribution. Given that Evo2 was trained on massive genomic datasets, high-likelihood sequences are more likely to adhere to the distributional grammar and statistical patterns observed in real genomic data.

Enformer We adapted the pretrained Enformer model from the PyTorch (`https://github.com/lucidrains/enformer-pytorch`) version by configuring the output bins to range from 1 to 896, using the central bins as the functional representation. We also filtered the output features (e.g., CAGE, histone modifications, accessibility, and transcription factor binding), resulting in a selection of 5,313 human features in total.

Sei We employed the official pretrained Sei model (`https://github.com/FunctionLab/sei-framework`), following the recommended Conda environment setup. We padded sequences to a length of 4096 bp to generate a vector of up to 21,907 dimensions. Subsequently, we directly filtered the features functionally associated with human. During feature analysis of the H-500 dataset, we found that the H3K9me3 features of two melanoma-related primary cells remained consistently high across sequences generated by different models as well as random sequences. We consider that this reflects a bias inherent in the models' training data. To ensure the reliability of our analysis, we therefore excluded these two features.

Borzoi We obtained four pre-trained Borzoi models (preprint stage) from `https://github.com/calico/borzoi` and established the environment using Conda and TensorFlow as recommended. By using the central bins as the functional representation, we selected a total of 7,611 human features, including CAGE, RNA-seq and ChIP-seq.

For Enformer, Sei, and Borzoi, sequences shorter than the required input length were padded with `N` nucleotides in the flanking regions before prediction. The same preprocessing was applied to both natural and generated sequences.

Analysis of BLAST search outcomes

We evaluated the similarity between our de novo designed nucleotide sequences and natural genomic sequences using the NCBI Basic Local Alignment Search Tool - Nucleotide (BLASTn) web interface (`https://blast.ncbi.nlm.nih.gov/Blast.cgi`). To ensure a clean and biologically meaningful comparison, we first excluded all

training datasets that contained synthetic or engineered (non-natural) sequences. This led to the removal of one *Escherichia coli* dataset (165bp).

For each of the remaining organism-specific datasets, the following procedure was applied:

- The standard nucleotide BLAST program (`blastn`) was selected with the "Highly similar sequences (megablast)" optimization.
- De novo designed or natural query sequences were uploaded in FASTA format.
- The search was restricted to the organism-specific subset of the core nucleotide collection (`core_nt`) using the corresponding taxonomy identifier:
  - *Escherichia coli* (taxid:562)
  - *Drosophila* (taxid:7215)
  - *Homo sapiens* (taxid:9606)
- The E-value threshold was set to 10,000 to accommodate the expected high dissimilarity between designed and natural sequences (a stricter threshold frequently returned zero hits).

For each organism, we submitted 100 sequences from the de novo design model outputs (e.g., BFN-generated sequences) and, for comparison, 100 natural sequences randomly sampled from the corresponding reference genome.

We subsequently analyzed the distributions of E-values and query coverage percentages across all significant alignments. The de novo designed sequences consistently showed very high E-values and low query coverage (Figure S1), comparable to those of completely random sequences, indicating low sequence similarity to natural genomic regions.

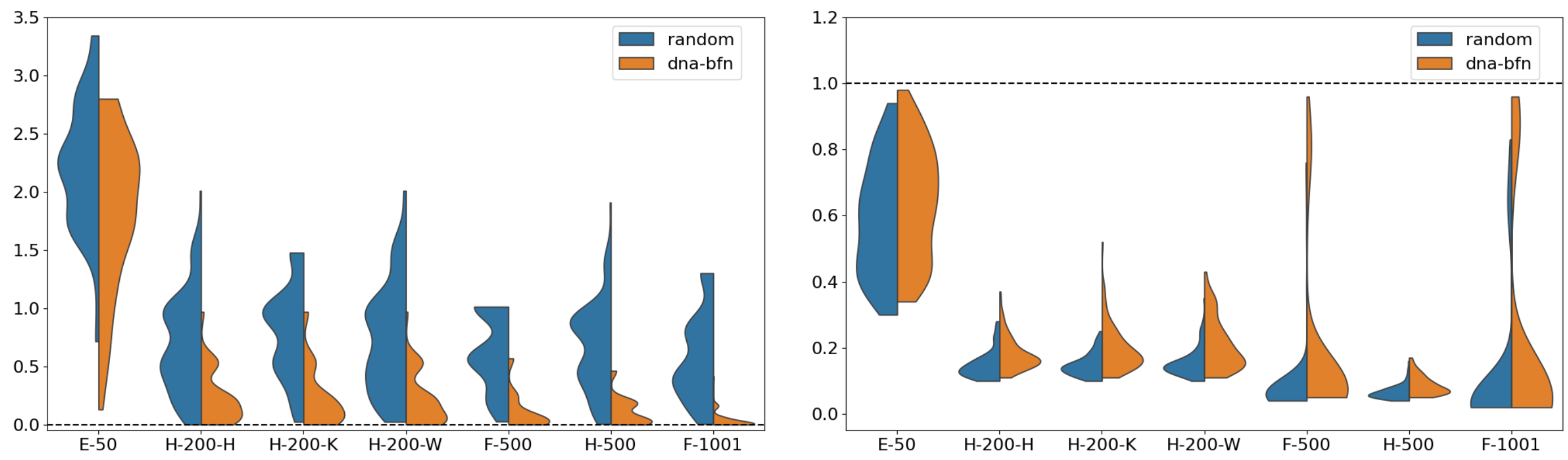

Left: E-value distribution (log10-transformed) **Right:** Query coverage distribution (%)

Figure S1 Distribution of BLASTn alignment metrics for de novo designed DNA sequences (DNA-BFN) compared to random sequences across tested organisms. Violin plots display E-values (left) and query coverage (right), with median (black) and mean (white) marked. The dashed lines indicate natural sequences in the corresponding training datasets, with E-values near 0 and coverage near 1.

Table S1 Comparison of k-mer frequency correlations for different generative models on the E-50 dataset.

Model	4-mer (↑)	5-mer (↑)	6-mer (↑)	7-mer (↑)	8-mer (↑)	9-mer (↑)
mdm	0.9547	0.9332	0.9006	0.8378	0.6910	0.3752
ddsm	0.9647	0.9544	0.9296	0.8759	0.7378	0.4243
wgan	0.5551	0.5096	0.4744	0.4339	0.3575	0.1794
pssm	0.7055	0.6606	0.6081	0.5283	0.3808	0.1284
promodiff	0.1754	0.1751	0.1700	0.1564	0.1274	0.0007
DNA-BFN	0.9834	0.9711	0.9442	0.8860	0.7441	0.4242

Table S2 Comparison of different regulatory sequence generation models across datasets based on mean intra-class edit distance.

Model	E-50	E-165	H-200-H	H-200-K	H-200-W	F-500	H-500	F-1001
mdm	22.57	79.89	98.86	98.52	97.17	256.09	253.94	518.89
ddsm	22.64	77.62	98.73	99.86	96.16	255.68	255.32	502.90
Wgan	21.05	69.70	90.53	94.65	88.25	246.99	230.58	470.20
pssm	23.06	79.89	99.38	98.68	97.04	252.76	253.29	505.19
promodiff	23.65	83.75	100.27	100.24	100.44	257.29	255.49	525.54
DNA-BFN	22.35	77.94	99.00	97.57	97.19	256.93	253.04	512.59

Table S3 Ablation study of DNA-BFN on the E-50 dataset. Performance is evaluated using GC content JS divergence, k-mer correlation (k=6), and average edit distance. Lower GC and ED values and higher K-MER values indicate better performance.

Model	GC ↓	K-MER ↑	ED ↓
DNA-BFN	**0.0045**	**0.9442**	**22.72**
w/o Bayesian Update	0.0063	0.8772	22.97
w/o Entropy Encoding	0.0167	0.8927	22.80
w/o Both	0.0193	0.8482	22.77

Table S4 Comparison of different regulatory sequence generation models on the H-500 dataset based on motif-pair occurrence, orientation, and spacing. A total of 1,191 motif pairs were selected from the H-500 training set for analysis. Values indicate the number of differential motif pairs compared with the training set, with lower values indicating better preservation of motif-level regulatory grammar.

Model	Occurrence	Orientation	Spacing
MDM	14	**6**	34
DDSM	7	9	30
WGAN	651	320	58
PSSM	370	12	52
PROMODIFF	51	9	47
DNA-BFN	**1**	**6**	**29**

Table S5 Benchmarking regulatory sequence generation models on the E-50 dataset using GC content JS divergence, k-mer correlation ($k = 6$), and average edit distance, evaluated on de-redundant training and test sets constructed using MMseqs2-based sequence clustering with an 80% sequence identity threshold.

Model	GC ↓	K-MER ↑	ED ↓
DNA-BFN	0.0119	**0.8440**	**22.70**
DDSM	0.0108	0.7865	22.89
MDM	**0.0075**	0.7512	22.75
PromoDiff	0.0714	0.1508	23.79
PSSM	0.0268	0.4762	23.00
WGAN	0.0710	0.2131	22.90

Algorithm S1 Training of DNA-BFN

Require: Training data $\mathcal{D}$, network Φ_ω, final precision β_1

Sample $\mathbf{x} \sim \mathcal{D}$ and $t \sim \mathcal{U}(0, 1)$

Sample noisy observation $\mathbf{y}^{(t)} \sim p_s(\mathbf{y}^{(t)} \mid \mathbf{x}; \alpha_t)$ ▷ see Eq. (2) in the main text

Compute posterior parameters $\boldsymbol{\theta}^{(t)} \leftarrow \mathrm{Softmax}\Big(\sum_{p=1}^{t} \mathbf{y}^{(p)}\Big)$

Predict $\boldsymbol{\phi}^{(t)} = \Phi_\omega\big(\mathrm{EntropyEncode}(\boldsymbol{\theta}^{(t)})\big)$

Compute loss $\mathcal{L} = \dfrac{\alpha(t)}{2} \big\|\mathbf{e}(\mathbf{x}) - \boldsymbol{\phi}^{(t)}\big\|^2$

Update ω by minimizing $\mathcal{L}$

Algorithm S2 Sequence generation with DNA-BFN

Require: Trained network Φ_ω, number of sampling steps T

Initialize $\mathbf{y}^{(0)} = \mathbf{0}$

for $i = 1, \ldots, T$ **do**

    $t \leftarrow (i-1)/T, \quad s \leftarrow i/T$

    Update posterior $\boldsymbol{\theta}^{(t)} \leftarrow \mathrm{Softmax}\big(\mathbf{y}^{(t)}\big)$

    Predict $\boldsymbol{\phi}^{(t)} = \Phi_\omega\big(\mathrm{EntropyEncode}(\boldsymbol{\theta}^{(t)})\big)$

    Sample noisy observation $\mathbf{y}^{(s)} \leftarrow \beta(s)\big(K\boldsymbol{\phi}^{(t)} - \mathbf{1}\big) + \sqrt{K\beta(s)}\,\mathbf{z}$, $\mathbf{z} \sim \mathcal{N}(\mathbf{0}, \mathbf{I})$ ▷ see Eq. (10) in the main text

end for

Sample $\mathbf{x}$ from $\mathrm{Categorical}\left(\mathrm{Softmax}(\mathbf{y}^{(1)})\right)$

return $\mathbf{x}$

References

V. Agarwal, F. Inoue, M. Schubach, D. Penzar, B. K. Martin, P. M. Dash, P. Keukeleire, Z. Zhang, A. Sohota, J. Zhao, et al. Massively parallel characterization of transcriptional regulatory elements. *Nature*, 639(8054):411–420, 2025.

Q. Du, M. N. Poon, X. Zeng, P. Zhang, Z. Wei, H. Wang, Y. Wang, L. Wei, and X. Wang. Synthetic promoter design in escherichia coli based on multinomial diffusion model. *Iscience*, 27(11), 2024.

S. J. Gosai, R. I. Castro, N. Fuentes, J. C. Butts, K. Mouri, M. Alasoadura, S. Kales, T. T. L. Nguyen, R. R. Noche, A. S. Rao, et al. Machine-guided design of cell-type-targeting cis-regulatory elements. *Nature*, 634(8036):1211–1220, 2024.

H. Wang, Q. Du, Y. Wang, H. Xu, Z. Wei, and X. Wang. Gpro: generative ai-empowered toolkit for promoter design. *Bioinformatics*, 40(3):btae123, 2024.
